# Altered splicing and cytoplasmic levels of tRNA synthetases in SF3B1-mutant myelodysplastic syndromes as a therapeutic vulnerability

**DOI:** 10.1038/s41598-019-39591-7

**Published:** 2019-02-25

**Authors:** Fabio Giuseppe Liberante, Katrina Lappin, Eliana M. Barros, Jekaterina Vohhodina, Florian Grebien, Kienan I. Savage, Kenneth Ian Mills

**Affiliations:** 1 Centre for Cancer Research and Cell Biology (CCRCB), Queen’s University Belfast, Belfast, United Kingdom; 2 Ludwig Boltzmann Institute for Cancer Research, Vienna, Austria; 3 Institute for Medicinal Biochemistry, University of Veterinary Medicine, Vienna, Austria

## Abstract

Myelodysplastic syndromes (MDS) are haematopoietic malignancies that are characterised by a heterogeneous clinical course. In recent years, sequencing efforts have uncovered recurrent somatic mutations within RNA splicing factors, including *SF3B1, SRSF2, U2AF1* and *ZRSR2*. The most frequently mutated gene is *SF3B1*, mutated in 17% of MDS patients. While *SF3B1* mutations and their effects on splicing have been well characterised, much remains to be explored about their more far-reaching effects on cellular homeostasis. Given that mRNA splicing and nuclear export are coordinated processes, we hypothesised that SF3B1 mutation might also affect export of certain mRNAs and that this may represent a targetable pathway for the treatment of *SF3B1*-mutant MDS. We used CRISPR/Cas9-genome editing to create isogenic cellular models. Comprehensive transcriptome and proteome profiling of these cells identified alterations in the splicing and export of components of the translational machinery, primarily tRNA synthetases, in response to the SF3B1 K700E mutation. While steady-state protein synthesis was unaffected, SF3B1 mutant cells were more sensitive to the clinically-relevant purine analogue, 8-azaguanine. In this study, we also demonstrated that 8-azaguanine affects splicing. Our results suggest that the simultaneous targeting of RNA metabolism and splicing by 8-azaguanine represents a therapeutic opportunity for SF3B1-mutant myelodysplastic syndromes.

## Introduction

Myelodysplastic syndromes (MDS) are myeloid haematopoietic malignancies that are characterised by an extremely heterogeneous clinical course, ranging from indolent disease without progression, to acute onset requiring immediate treatment. The variable course of MDS is mainly driven by molecular heterogeneity. There are many genetic defects associated with different prognoses, including numerous deletions and chromosomal losses^1^, implying that the dysplasia of MDS is generally due to loss or alteration of gene function.

In recent years, genome and exome-wide sequencing studies in MDS have uncovered a number of recurrent somatic mutations within mRNA splicing factors, including *SF3B1, SRSF2, U2AF1* and *ZRSR2*. The most frequent of these were in *SF3B1*, representing 17% of all mutations in MDS^2^, which in isolation generally infer a better prognosis. This is likely due to their high frequency in the refractory anaemia with ringed sideroblasts (RARS) subtype of MDS, which has a clinically more benign phenotype, with a lower likelihood of transformation. Indeed, mutations of *SF3B1* occur in up to 90% of patients with RARS and in 70% of those with refractory cytopenia with multilineage dysplasia and ring sideroblasts (RCMD-RS). The presence of ringed sideroblasts, which arise from abnormal iron deposits, was recently demonstrated to be directly related to the presence of *SF3B1* mutations^3^. At the molecular level, mutant SF3B1 results in abnormal splicing of several genes, primarily due to misrecognition of 3′ splice sites^4^. Many of the resulting aberrant mRNAs undergo nonsense-mediated mRNA decay (NMD), leading to reduced gene expression. This was shown to affect many genes important for iron metabolism in haematopoietic cells, which likely explains the iron transport defects observed in these cells^5,6^.

While the connection between *SF3B1* mutations and its effects on splicing at the molecular level has been well characterised^7^, much remains to be explored about its more far-reaching effects on cell homeostasis. It has been known for many years that mRNA splicing and nuclear export are coordinated processes, that are tightly-linked^8–10^. More recent research has begun to demonstrate a direct connection between alternative splicing and cytoplasmic abundance of transcripts as a mechanism of control^11,12^. Therefore, we hypothesised that SF3B1, being a critical part of the spliceosome, might also affect cytoplasmic levels of mRNA species. We sought to investigate whether this role of SF3B1 represented a strategy for targeting mutant cells for clinical benefit.

Our data propose that SF3B1 mutations lead to defects in the splicing and export of mRNAs encoding components of the translational machinery. While steady-state protein synthesis appears unaffected, SF3B1 mutant cells were more sensitive to the clinically-relevant purine analogue, 8-azaguanine. This sensitivity suggests that simultaneous targeting of both RNA metabolism and splicing by this single compound represents a therapeutic opportunity for patients suffering from SF3B1 mutant myelodysplastic syndromes.

## Results

### CRISPR/Cas9-edited cells express K700E mutant SF3B1 at equivalent mRNA and protein ratios

Whilst a few cell lines harbouring *SF3B1* mutations do exist, none is derived from haematopoietic tissues. Therefore, to study the effects of the SF3B1 K700E mutation in isolation, we set out to create isogenic models of this mutation in haematopoietic cell lines. K-562 cells were edited using CRISPR/Cas9 and single-stranded oligodeoxynucleotides (ssODN) to introduce an A > G substitution in codon 700 of the *SF3B1* gene, the mutation observed in the majority of MDS patients. A synonymous, tracking mutation was also introduced at codon 701, creating a new MspI restriction site (Fig. S1A).

**Figure 1:**
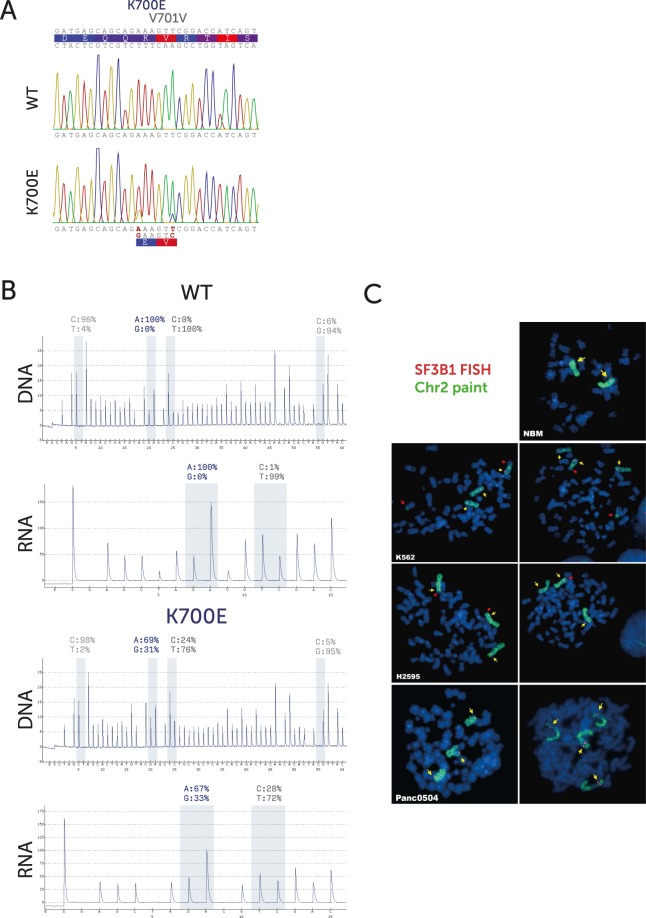
(**A**) Sanger sequencing of the targeted genomic region from both wildtype and K700E mutated K-562 cells. Double chromatogram peaks representing different nucleotides are labelled in red. (**B**) **(DNA)** Pyrosequencing of the targeted genomic region from both wildtype and K700E mutated K-562 cells. The calculated allelic ratio is displayed for both the A > G (K700E) and T > C (V701V) nucleotides. The other ratios in light grey represent control reactions that ideally yield zero. **(RNA)** Pyrosequencing of cDNA via RT-PCR representing the ratio of RNA species for the same nucleotides. (**C**) Fluorescent *in situ* hybridization (FISH) of metaphase spreads from normal lymphocytes (NBM), H-2595 (K700E), Panc0504 (K700E) and K-562 cells. Blue – DAPI; Green – Whole chromosome 2 paint; Red - Fosmid G248P85642F7 [SF3B1]. All A minimum of 100 cells scored per cell line. All samples showed ≥3 signals for SF3B1 in ≥85% of cells.

Successful editing of the locus was identified through restriction fragment length polymorphism (RFLP), as digestion by MspI would create two fragments instead of one (Fig. S1B). Sanger sequencing of successfully edited cells showed a double peak at both the K700E A > G and V701V T > C nucleotides (Fig. 1A). Pyrosequencing of DNA and RNA showed that approximately 30% of both DNA and RNA reads contained the mutant A > G allele (Fig. 1B). These mutated cells are henceforth designated SF3B1^K700E^.

Our initial attempts to target a number of other MDS and AML cell lines, including OCI/AML-3 and MDS-L, failed. Given that the mutant allele burden in our modified cells deviated significantly from 50%, and an anecdotal finding by Zhou *et al*.^13^, we hypothesised that it may have been due to these cell lines only harbouring two copies of SF3B1. Therefore, we used FISH to assess SF3B1 copy number in these cell lines. This revealed that K-562 cells carry at least 3 copies of chromosome 2 and, accordingly, 3 copies of the SF3B1 locus (Fig. 1C). Similarly, testing of two other human cancer-derived cell lines that carry SF3B1 K700E mutations (H-2595 and Panc0504) identified three or more copies of SF3B1. The finding that all reported SF3B1-mutant cell lines (including ESS-1) carry three copies of chromosome 2 may reflect a requirement of immortal cell lines for a lower dosage of the mutant protein.

Further validation of the mutant clones by T/A cloning of the locus agreed with the FISH and pyrosequencing data, showing that mutant cells possessed the A > G transition on only one of three alleles (data not shown). One clone, negative by RFLP and designated SF3B1^+/Δ/Δ^, carried two different out-of-frame deletions; retaining only a single functional copy of SF3B1 (Fig. S1C).

In order to verify that the mutated allele was translated into protein, SF3B1 was immunoprecipitated (using an N-terminal antibody) from wildtype and SF3B1^K700E^ cells (Fig. S1D) and analysed by mass spectrometry. As the K700E mutation destroys a tryptic cleavage site in SF3B1, we were able to confirm the presence of the glutamic acid-containing peptide in the successfully mutated clone. In accordance with mRNA expression levels, label-free quantitation of peptide fragments estimated that between 32% and 46% of the immunoprecipitated SF3B1 protein contained the K700E substitution. This implies that the K700E change does not drastically affect the translation or stability of the protein.

### SF3B1^K700E^ has similar effects on splicing in primary cells and cell lines

We next set out to determine if SF3B1 mutation led to altered mRNA-splicing in this model system and whether this altered splicing reflected the patterns that are seen in MDS patients and other models of the same mutation. Therefore, publically available RNA-Seq data from CD34^+^ bone marrow cells from MDS patients^14^ and NALM-6 cell line models^4^ were re-analysed and directly compared.

**Figure 2:**
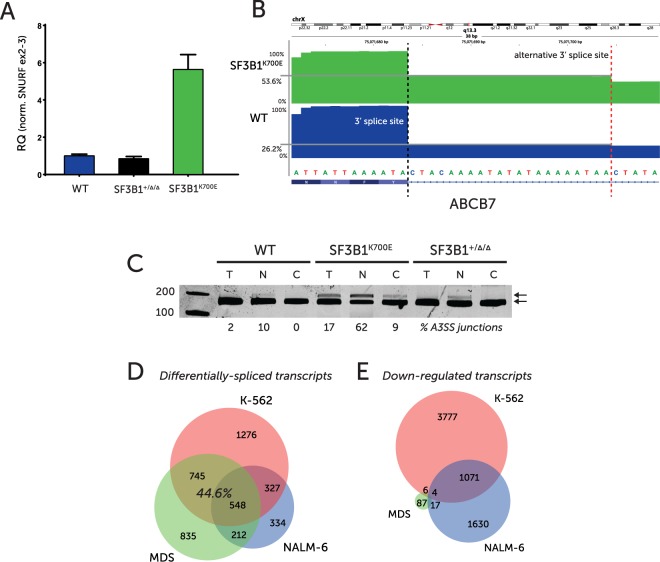
(**A**) Relative quantities of SNURF exon 2b measured by qPCR across ex2-2b and normalized to ex2-3 levels. Error bars represent SEM. (**B**) RNA-Seq, total read count-normalized, coverage plots at the ABCB7 exon previously published as having an alternative 3′ splice site. Note: gene is on reverse strand. (**C**) RT-PCR of cDNA from wildtype, SF3B1^K700E^ & SF3B1^+/Δ/Δ^ whole cell (T), nuclear (N) and cytoplasmic fractions (**C**). Amplicon spans ABCB7 exons 8–9 (RefSeq NM_004299). Percentages represent RNA-seq reads that span the alternative 3′ splice site as a fraction of the total number of reads spanning exons 8 and 9. (**D**) Venn diagram representing overlap in genes identified as having altered splicing by dSpliceType (all types) in SF3B1^K700E^ vs wildtype samples. Comparing RNA-Seq data from Darman *et al*. (NALM-6 cells, n = 3), Dolatshad *et al*. (primary MDS, n = 4) and this study (n = 2). (**E**) Venn diagram representing overlap in genes identified as downregulated at the transcript-level by DESeq. 2 analysis in SF3B1^K700E^ vs wildtype samples. Comparing RNA-Seq data from Darman *et al*. (NALM-6 cells), Dolatshad *et al*. (primary MDS) and this study.

Initial analysis identified strong inclusion of a cryptic exon within the *SNURF/SNRPN* gene in SF3B1 mutant cells in both public data sets. We determined whether our SF3B1^K700E^ modified cells showed similar inclusion of this cryptic exon ‘2b’ by qPCR. We found significantly higher expression levels of the cryptic exon ‘2b’ in the SF3B1^K700E^ clone compared to wildtype or +/Δ/Δ cells (Fig. 2A). To enable more comprehensive global comparisons, we performed total RNA-Seq on our isogenic cell line model. Encouragingly, increased alternate 3′ splice site usage within the *ABCB7* gene (chrX:75,071,693-75,071,704 - hg38) was observed in the SF3B1^K700E^ cells (Fig. 2B,C), in agreement with what has been identified by a number of groups^4,6^.

Investigation of differentially spliced transcripts identified significant overlaps between the model generated in this study and those previously published. In particular, 44.6% of genes that were identified as differentially-spliced in our isogenic model were also altered in primary MDS samples with *SF3B1*^K700E^ mutation (Fig. 2D). This suggests a degree of consistency in the effects of *SF3B1*^K700E^ on the splicing of particular genes, despite different cellular contexts. Thus demonstrating the relevance of cell lines as models to study this mutation and validating our isogenic model of the *SF3B1*^K700E^ mutation to physiologically represent changes in RNA splicing and expression as seen in primary patient material.

Interestingly, the majority of differential events in all comparisons were skipped exons, representing more than 45% of total events in this analysis of the RNA-Seq data (Table 1). This suggests an overall disruption of exon recognition, despite the more commonly described effect of SF3B1^K700E^ on 3′ splice site recognition^15^. In contrast to the splicing analysis, only 6 down-regulated transcripts overlapped between this study and primary MDS samples (Fig. 2E). This illustrates the limitations of whole-transcript level quantitative RNA-Seq in comparing the effects of spliceosome mutations on the transcriptome.

**Table 1:** Differentially spliced or expressed genes for data sets analysed.

Type of alteration WT vs K700E	MDS^6^	NALM-6^4^	This study (whole cell)
A3SS	187	262	405
A5SS	207	180	362
MXE	99	46	249
RI	937	323	580
SE	1385	834	2111
*Total unique changes*	2815	1421	2896
Up-regulated	156	72	1566
Down-regulated	132	89	1386

Numbers represent unique genes. Differential expression threshold - fold-change >2 & FDR < 0.05 A3SS/A5SS – alternative 3′/5′ splice site; MXE – Mutually exclusive exons; SE – Skipped exons; RI – Retained introns.

On a functional level, enrichment analysis (using Enrichr) of the 548 common differentially-spliced genes in all three data sets (Fig. 2D) identified the ribosome, RNA transport and spliceosome pathways (Table 2), as significantly altered in SF3B1^K700E^ cells.

**Table 2:** Enrichr – KEGG 2016 pathway enrichment from 548 genes identified as differentially-spliced in all three studies; primary MDS, NALM-6 cell line & this study.

Term	Overlap	Adjusted P-value	Z-score	Combined Score
Ribosome - hsa03010	15/137	0.0012	−1.75	21.16
RNA transport - hsa03013	15/172	0.0063	−1.84	17.35
Spliceosome - hsa03040	13/134	0.0063	−1.75	16.44
mRNA surveillance pathway - hsa03015	10/91	0.011	−1.64	14.05
Metabolic pathways - hsa01100	53/1239	0.040	−1.91	13.47

### SF3B1^K700E^ leads to decreased levels of cytoplasmic mRNA

**Figure 3:**
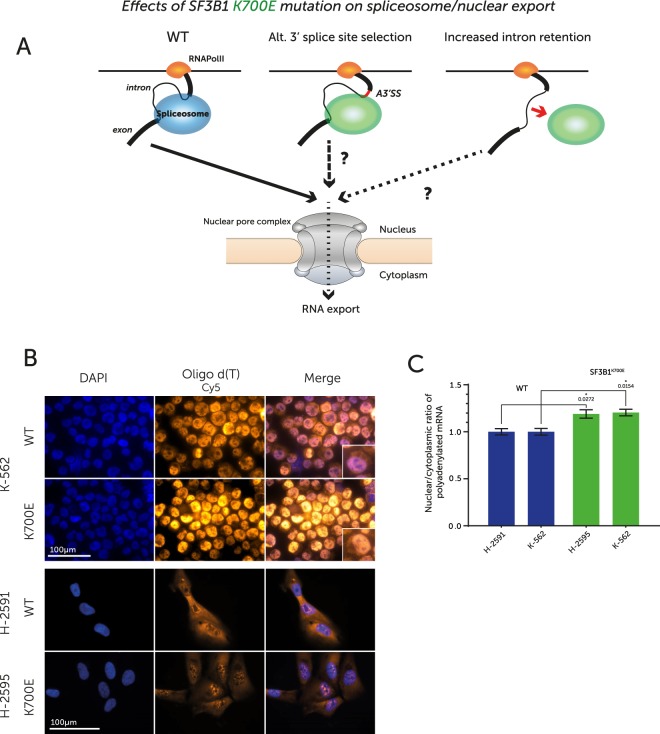
(**A**) Schematic illustrating the effects of SF3B1 mutation on splicing and predicted roles in mRNA export. (**B**) FISH of cells fixed and stained with DAPI and Oligo d(T)_50_-Cy5. H-2595 (SF3B1^K700E^) & H-2591 (SF3B1^WT^) cells were grown directly on slides. K-562 cells were spun onto slides. Images for the same fluorophore were captured using identical exposure times and gain. Raw monochrome images colorized using ImageJ and overlaid in the final panels. (**C**) A plot representing the nuclear to cytoplasmic ratio of the Oligo d(T)_50_-Cy5 signal. Multiple FISH images were captured from replicate stainings and cytoplasmic Cy5 signal was quantified as a ratio of nuclear signal using an image reduction algorithm written in ImageJ. WT values set as 1. Error bars represent SEM.

Numerous studies have demonstrated a tight link between RNA splicing and export, demonstrating that efficient splicing is required before RNA export can occur^16–18^. To examine whether SF3B1^K700E^ expression had a global effect on mRNA export (Fig. 3A), we used polyA-targeting FISH to localise total mRNA species in both our isogenic K-562 cells and two similar mesothelioma cell lines (Fig. 3B). Quantification revealed that those cells harbouring SF3B1^K700E^ displayed a significant increase (~20%) in nuclear mRNA staining intensity (Fig. 3C) compared to their SF3B1^WT^ counterparts, suggesting a defect in mRNA export.

Based on these observations, we set out to examine if SF3B1^K700E^ expression was associated with reduced cytoplasmic levels of specific mRNAs. We isolated cytoplasmic and nuclear RNA from WT and SF3B1^K700E^ expressing cells. To assess the purity of the samples, we quantified the levels of the nuclear lncRNAs MALAT1 and NEAT1^19^, which were ≥30-fold enriched in nuclear vs. cytoplasmic fractions (Fig. S2A). Subsequent RNA-seq and data analysis confirmed a much greater coverage of MALAT1 and NEAT1 in nuclear RNA fractions compared to cytoplasmic fractions (Fig. S2B). The small nucleolar RNA (snoRNA) SNORD47 was also highly enriched in the nuclear fraction (Fig. S2B).

**Figure 4:**
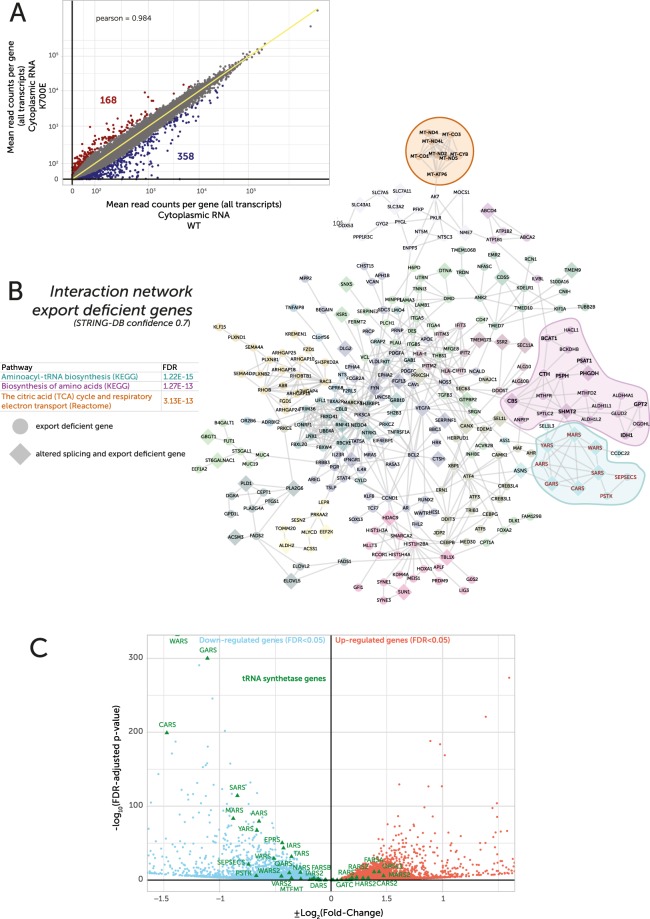
(**A**) Scatter plot of mean cytoplasmic RNA-Seq read counts per gene, as normalised by DESeq2, comparing K700E cells to wildtype cells (n = 2). Axes plotted as biexponential (red = FDR < 0.05 FC > 2, blue = FDR < 0.05 & FC < −2). (**B**) Network of directly interacting proteins based on the set of genes identified as depleted in the cytoplasmic fraction of mutant cells (FDR0.05 FC < −1.5). Transcripts identified as having altered splicing in mutant cells are indicated as diamonds. Genes belonging to tRNA synthesis pathway (KEGG hsa00970) are labelled in red. Nodes coloured according to cluster assigned by Reactome FI. Protein-protein interactions identified by STRING-DB. Table lists top three enriched pathways for highlighted gene clusters and their FDR-adjusted p-values. (**C**) Plot of –log_10_(FDR-adjusted p-value) against log_2_(fold-change) for cytoplasmic RNAs WT vs SF3B1^K700E^ –only log2(fold-change) ±1.5 shown (n = 2). Genes from tRNA synthetase pathway (KEGG hsa00970) plotted as green triangles. Significantly differentially-expressed tRNA synthetases are labelled.

RNA-Seq analysis revealed that a higher number of genes were decreased in the cytoplasmic RNA fraction of SF3B1 mutant cells than increased (358 vs 168 respectively) (FDR < 0.05 & FC ± 2 | Fig. 4A). An interaction network of the under-represented genes (FDR < 0.05 & FC < −1.5 | Fig. 4B) illustrates that not all of the export deficient transcripts are actually mis-spliced. This suggests that the SF3B1-related export defect is not always related to altered splicing, but may reflect a temporal constraint on co-ordinated splicing and export. The specific clusters of genes identified as being down-regulated include components of Aminoacyl-tRNA biosynthesis (blue cluster), Biosynthesis of amino acids (mauve cluster) and the TCA cycle (orange cluster). Among the mis-spliced/lesser-exported genes in the network (49 genes) the pathways Aminoacyl-tRNA biosynthesis (KEGG hsa00970) and Cytosolic tRNA aminoacylation (Reactome HSA-379716) were highly enriched (red labels). Sequence analysis of tRNA synthetase splicing junctions that were altered in SF3B1^K700E^ cells shows that they frequently possess an AG-GT donor-acceptor consensus site at position −28 (Fig. S2C). Strikingly, we found that 21 of the 44 human tRNA synthetases (48%) were significantly depleted in the cytoplasmic RNA fraction of SF3B1 mutant cells (Fig. S2D | Fig. 4C). Validation by qPCR of tryptophanyl-tRNA synthetase (WARS) showed accumulation of nuclear transcripts, but an almost 20% reduction at the cytoplasmic level (Fig. S2E). Taken together, this implies a deficiency in tRNA synthesis, caused by the defective splicing and/or export of crucial transcripts in SF3B1 mutant cells.

### SF3B1^K700E^ leads to reduced protein levels of components of the translational machinery and tRNA metabolism, but to an increase in spliceosome components

**Figure 5:**
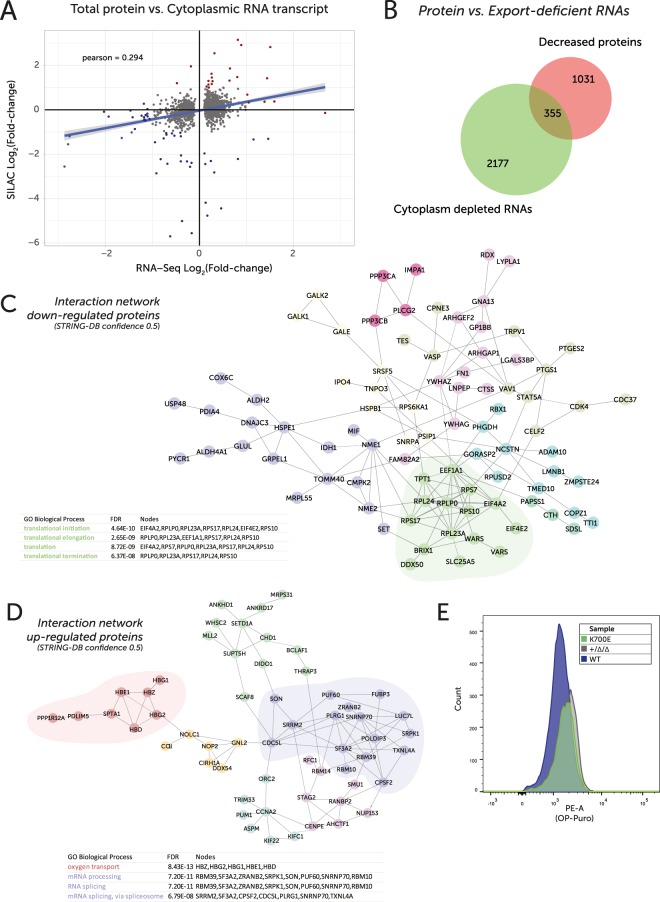
(**A**) Scatter plot of log2-transformed protein fold change (SILAC, n = 1, 2702 unique protein hits passing QC) vs cytoplasmic transcript log2-transformed fold change (RNA-Seq - DESeq. 2, n = 2). Pearson correlation = +0.294. Red = FC > 2 | Blue = FC < −2 in both comparisons. (**B**) Venn diagram representing overlap in genes identified as being depleted in the cytoplasm (FDR < 0.05) and down at the protein level by SILAC, comparing SF3B1^K700E^ vs wildtype samples. (**C**) Network of directly interacting proteins based on those identified as being decreased in the SF3B1^K700E^ cells by SILAC. Nodes coloured according to cluster assigned by Reactome FI. PPIs identified by STRING-DB (direct, 0.5 confidence). Table identifies top 4 significantly enriched gene ontology terms from Reactome, all related to translation (green cluster). (**D**) Network of directly interacting proteins based on those identified as being increased in the K700E cells by SILAC. Nodes coloured according to cluster assigned by Reactome FI. PPIs identified by STRING-DB. Table identifies top 4 significantly enriched gene ontology terms from Reactome, including oxygen transport (red) and splicing (purple). (**E**) Flow cytometry histograms of cellular fluorescence representing OP-Puromycin (OP-Puro) uptake in wildtype, +/Δ/Δ and +/+/K700E cells.

Given the changes in mRNA splicing and export observed in the SF3B1^K700E^ cells, we set out to determine the impact of these changes on protein expression. To investigate the effects of the *SF3B1*^K700E^ mutation on the proteome, we used Stable Isotopic Labelling by Amino acids in Culture (SILAC). This quantitative mass spectrometry approach identified a number of proteins whose steady-state levels were altered significantly in SF3B1^K700E^ cells compared to wildtype cells. While there was no correlation between transcript and protein levels at the whole cell level (Fig. S3A), changes between cytoplasmic transcript and whole cell proteome showed a positive correlation (Pearson = +0.294 | Fig. 5A). This is also reflected in the divergence of some transcripts from a general correlation between the three subcellular fractions (Fig. S3B).

Intersection of RNA-seq and SILAC-MS datasets showed that 355 genes had both decreased cytoplasmic levels and reduced protein expression upon SF3B1 mutation (Fig. 5B). Indeed, 10 of the 21 tRNA synthetases that showed significantly reduced cytoplasmic mRNA levels, were also decreased at the protein level (Table S9). Moreover, an interaction network of the proteins down-regulated upon SF3B1 mutation shows enrichment for a cluster of genes related to translation (Fig. 5C), the same pathway enriched in all cytoplasm-depleted genes (FDR < 0.05). The translation machinery was found among the most strongly down-regulated proteins (>3-fold) with some ribosomal proteins (RPS/L) and elongation initiation factors (EIF) decreased at the protein level by more than 10-fold (Table S5).

In contrast, the most significantly enriched process among an interaction network of proteins upregulated in the SF3B1^K700E^ cells (Fig. 5D) was oxygen transport (GO:0015671). This represents the haemoglobin chains (red cluster) — of which 4 subunits were increased >5-fold. In keeping with this, cell pellets from SF3B1 mutant cells were consistently redder in colour, reflecting increased haemoglobin levels (Fig. S4A). Components of the RNA splicing machinery were also enriched among up-regulated proteins (purple cluster), suggesting a possible feedback loop as the cell attempts to compensate for global mis-splicing.

Despite a significant reduction in a number of key ribosomal components at protein, mRNA and tRNA levels, SF3B1^K700E^ cells did not show obvious defects in steady-state protein synthesis, as measured via similar rates of OP-Puro incorporation into the ribosome (Fig. 5E). Further, the clone possessing only a single functional copy of SF3B1 (+/Δ/Δ) also displayed similar rates of protein synthesis, suggesting that SF3B1 copy number alone does not influence global protein synthesis rates. Concomitantly, these data imply that SF3B1 mutation leads to depletion of tRNA pools through reduced tRNA synthetase levels, combined with a deficiency in translational machinery components, without drastically affecting steady-state protein synthesis in non-stressed cells.

### SF3B1 mutation increases sensitivity to RNA analogs

**Figure 6:**
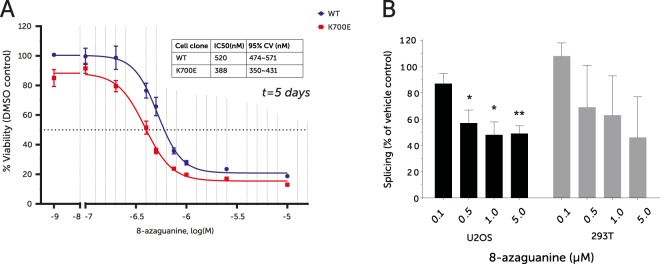
(**A**) Dose-response curve of 8-azaguanine treatment for 5 days; plotted as % viability as ATP levels normalised to vehicle-treated controls. The table represents calculated 50% inhibitory concentrations (IC_50_) and 95% confidence values (n = 3, technical duplicates). (**B**) Percentage of cells showing wildtype level of splicing, as measured by ratio of red to green fluorescence, after treatment with the indicated concentration of 8-azaguanine. At least 100 cells scored by high-content analysis.

Our data show that SF3B1^K700E^ cells have defects in the splicing and cytoplasmic export of tRNA synthetases and RNA metabolism-related factors. Given this, together with the apparent decrease in ribosomal proteins, we hypothesised that SF3B1^K700E^ cells might be hypersensitive to drugs targeting a compromised translational machinery. The nucleoside analogue 8-azaguanine is known to incorporate into rRNA and tRNA, and can block regular guanine incorporation^20–22^. This artificial base leads to the production of non-functional RNA species, thereby interfering with ribosome function. Thus we hypothesised that 8-azaguanine may selectively target SF3B1^K700E^ cells over ribosome/translation replete wildtype cells. Indeed, SF3B1^K700E^ cells were more sensitive to treatment with varying doses of 8-azaguanine (Fig. 6A).

As 8-azaguanine is also incorporated into mRNA species and as guanine is the most frequent base that is cleaved before transesterification in the predominant GT/AG cleavage sites, we suspected that its selective effect on SF3B1 mutant cells may also be due to an effect on mRNA splicing. To quantify 8-azaguanine-induced changes in correct intron removal, we used an exon-intron-exon two-colour fluorescent splicing reporter. Treatment of cell lines U2OS and 293-T harbouring this reporter with 8-azaguanine led to a decrease in relative fluorescence levels and therefore correct splicing, suggesting that 8-azaguanine does inhibit normal mRNA-splicing functions (Fig. 6B).

## Discussion

As a health burden, myelodysplastic syndromes represent a considerable proportion of haematological malignancies, constituting 5.7% of total incidence^23^. Considering that the median age at diagnosis is 75.8 years, the use of high-intensity chemotherapy regimens is often not possible due to the lack of fitness of these patients to tolerate such therapies, so novel therapeutic options are desperately needed. The discovery of spliceosome mutations in myelodysplastic syndromes has led to an explosion of research in this field. Indeed, screening for these mutations has identified their presence in many other cancers, including chronic lymphocytic leukaemia (CLL)^24^, uveal melanoma^25^ and breast cancer^26^. While a number of novel compound classes have been discovered that target the spliceosome machinery directly, such as Spliceostatin A, Pladienolides and more recently H3B-8800, these drugs are far from clinical application and some have even proven difficult to synthesise^27–29^. Therefore, alternative means of selectively targeting the SF3B1 mutated cells in patients would be of significant clinical benefit.

There already exist a multitude of recent publications, which investigate the effects of SF3B1 mutations on splice-site selection^4,7,15,30^. Many of these studies have demonstrated that mutant SF3B1 leads to differential 3′ splice site preference during splicing. Another recent paper also investigated altered splicing as a signature of SF3B1 mutations, showing that it led to a pattern distinct from other spliceosome mutations^31^. In this study, we aimed to identify the downstream effects of SF3B1^K700E^ mutation on cellular homeostasis through modelling of isogenic cell lines.

The most immediate effect of introducing the K700E mutation was a change in cell colour, which was reflected by an increase in foetal haemoglobin proteins in the SF3B1 mutant clone. This mirrors other research revealing alterations in haemoglobin synthesis in MDS patients^32^. Recent screening in healthy elderly populations has identified clonal haematopoiesis of indeterminate potential (CHIP) with an increase in the frequency of spliceosome mutations in haematopoietic stem cells^33^. While a relatively new concept, some hypothesise that CHIP may expand a failing stem cell pool, helping maintain sufficient levels of red blood cells^34–36^. Therefore SF3B1^K700E^, despite its association with malignant diseases, may initially be beneficial in increasing haemoglobin levels during age-related CHIP.

In comparing our genome-edited cells to existing data sets, we identified the cryptic exon ‘2b’ in the *SNURF/SNRPN* gene as strongly preferred in all SF3B1^K700E^ samples considered in this study. This may represent a more sensitive surrogate biomarker for SF3B1 mutation in either the clinic or the research laboratory, where clonal diversity might mask small minor allele frequencies.

Given the intimate relationship between splicing and nuclear export, we hypothesised that the SF3B1 mutation might lead to a defect in RNA export. While analysis of cytoplasmic RNA-Seq supported this hypothesis, it also showed that the effect was not uniform for all transcripts. Reassuringly, the RNA-Seq data from all three fractions (total, nuclear, cytoplasmic) correlate reasonably well between wildtype and SF3B1-mutant cells. In contrast, the abundances of tRNA synthetase genes were inversely correlated between whole cell extracts and nuclear/cytoplasmic fractions. This suggests that lower levels of certain cytoplasmic RNAs may not simply be due to decreased export, but also decreased transcription in the nucleus or increased exclusion from efficient translation, perhaps by sequestration into processing bodies (P-bodies). In line with this, we demonstrated cytoplasmic transcript levels correlate with actual protein levels better than total transcript levels, as many mRNAs have recently been shown to be only repressed in P-bodies and not decayed^37^. It has also been shown that other components of the nuclear export machinery (NXF1/XPO1) are also mutated in CLL, mutually-exclusive of SF3B1 mutations, suggesting that altered export may phenocopy SF3B1 mutation^38^. However, caution should be used when comparing RNA-Seq quantification between different cellular compartments, as it is known that different fractionation methods can yield different estimated transcript levels^39^. Therefore, we focused our downstream enrichment analysis on the cytoplasmic RNA levels.

The most interesting finding was a defect in the splicing and export of mRNAs encoding tRNA synthetases and ribosomal components. Additionally, we observed decreased expression of alpha-aminoacyl-tRNA binding proteins in the SILAC-MS data, concomitant with tRNA depletion. While direct quantification of tRNAs is difficult in regular RNA-Seq datasets, this warrants further investigation. Despite these changes, steady-state levels of protein synthesis remained unaffected in SF3B1 mutant cells, presumably through up-regulation of compensatory proteins, such as BCLAF1 and SON, which have roles in ribosome biogenesis and mRNA processing^40^. These proteins may have been upregulated to meet the increased demand of proliferative capacity and thus become required to maintain high proliferation rates in SF3B1 mutant cells making them attractive targets for splicing-deficient neoplasms.

The relationship between tRNA maintenance and MDS is not entirely new. Sideroblastic anaemia has been connected to a mitochondrial tRNA defect in a rare mitochondrial disorder known as mitochondrial myopathy, lactic acidosis and sideroblastic anemia (MLASA) syndrome^41^. Interestingly, one of the functional clusters in the network of export-deficient transcripts includes MT-ATP6, whose mutation was associated with MLASA^42^. Furthermore, there is a difference in tRNA levels in MDS compared to healthy haematopoietic tissue^43^. However, these tRNA defects have not yet been associated with altered SF3B1 status.

We theorized that the deficiency in tRNAs and ribosomal components might represent a chemically-tractable target in SF3B1 mutant cells. The synthetic nucleoside analogue 8-azaguanine has been investigated for over 70 years^44^ and has been reported to have anti-tumour activity in mouse models^45^. More recent data has shown that it is able to induce differentiation in leukaemia cell lines, an effect that was likely mediated through its incorporation into tRNA species^46^. We found that the SF3B1 mutant cells were more sensitive to this compound when compared to cells with wild-type SF3B1. While the high doses of 8-azaguanine that were needed for efficacy against leukaemia as a single agent led to side effects, it might be used at significantly lower dose in MDS, a much more indolent disease, which typically requires lower doses of chemotherapy.

In addition to its well-established effect on tRNA function, our data also show that 8-azaguanine leads to a defect in mRNA splicing. Therefore, SF3B1 mutations leading to simultaneous defects in both splicing and tRNA/ribosome stability might expose a unique vulnerability for dual-targeting using 8-azaguanine. Additionally, 8-azaguanine does not cross the blood-brain barrier nor is it intercalated or incorporated into DNA. This reduces the risk of cerebellar toxicity or therapy-induced leukaemias, which often result from DNA-damaging chemotherapies, such as cytarabine and daunorubicin. The dual-targeting effect of 8-azaguanine may also partially explain the greater effectiveness of azacytidine in SF3B1 mutant MDS patients as it is also incorporated into RNA/tRNA^47,48^.

In conclusion, using isogenic cell systems, we show that the SF3B1^K700E^ mutation induces consistent alterations in splicing regardless of cellular context. Additionally, it creates a defect in the nuclear export of polyadenylated mRNAs and a dysregulation of tRNA and ribosome homeostasis. Based on its ability to target both tRNA metabolism and splicing, we propose a new therapeutic application for the riboside analogue 8-azaguanine in treating patients harbouring SF3B1 mutations, such as breast cancer, uveal melanoma, myelodysplastic syndromes, acute myeloid leukaemia and beyond.

## Materials and Methods

### Published data analysis

RNA-Seq data from SF3B1 mutant NALM-6 cells was published by Darman *et al*.^4^ (NCBI PRJNA295064; GEO: GSE72790)^4^. RNA-Seq data from MDS patients and healthy donors was published by Dolatshad *et al*. (NCBI PRJNA268220; GEO: GSE63569)^14^. Both datasets were downloaded as raw FASTQ reads and processed using the same pipeline.

### Cell lines

K-562 cells, obtained from DSMZ, were maintained in RPMI-1640 (Sigma Aldrich), supplemented with FBS Superior (Biochrom AG) and 1% Penicillin/Streptomycin (Gibco) at 37 °C 5% CO_2_. In the case of long-term culture for dilution cloning, complete media was supplemented with 2 mM HEPES (Sigma-Aldrich) and 1 mM UltraGlutamine I (Lonza Biochem).

### Plasmids

The pSpCas9(BB)-2A-GFP (PX458) plasmid was a gift from Feng Zhang^49^ (Addgene plasmid # 48138). The pmKaxxte plasmid (now available as Addgene plasmid #113630), used to check gRNA efficiency, was generated using the pmaxGFP vector (Lonza) as a backbone and the mKate2.5-C1 — a gift from Michael Davidson (Addgene plasmid # 54828) — to generate overlapping insert fragments and a multiple cloning site.

The RFP2GFP-ATM construct was generated by inserting RFP sequence from mKate2.5-C1 into the pmaxGFP vector between KpnI and AgeI sites. The sequence encoding the ATM exon20-intron20-exon21 was PCR amplified from 293 T derived genomic DNA using primer sequences in Table S1 and cloned into the vector’s ORF between RFP and GFP tags using HindIII and SalI enzymes.

### CRISPR/Cas9

Guide RNAs (gRNA) were chosen using the online tool designed by Zhang *et al*. ssODNs were designed that contained the desired substitutions together with 77 bp of homology either side of the targeted substitutions. The gRNAs were tested using the mKaxxte plasmid (Addgene #113630) containing a cloned fragment of the intended target genomic region. This assessed the effectiveness of DSB creation by the gRNA by triggering HR-repair of the template to re-establish fluorescence. The final gRNA chosen yielded bright fluorescence in the majority of control cells, compared to those without the gRNA, which showed no background fluorescence (Fig. S4C).

The ssODN and gRNA/SpCas9-expressing plasmid (gRNA cloned into Addgene #48138) were nucleofected into K-562 cells. Cells were then sorted to purity for GFP-positivity and sub-cloned by dilution. The mutant allele burden and mRNA expression level was checked by Pyrosequencing.

### FISH & T/A cloning

FISH analysis was performed at the Wellcome Centre for Human Genetics, Oxford. For polyA FISH, cells were stained with fluorescent Oligo(d)T_50_ probes, as previously described^16^.

The targeted genomic region was amplified by PCR and then T/A cloned. At least 9 clones from each sample were sequenced.

### Proteomics

For SILAC, cells were grown in either “light” or “medium” RPMI and crude lysates analysed by FingerPrints Proteomics, Dundee. Data were analysed using MaxQuant software. For protein synthesis measurements, the EZClick™ Global Protein Synthesis Assay Kit (BioVision Inc., USA) kit was utilised.

### Splicing reporter assay

In the assay, a reporter plasmid encodes green and red fluorescent proteins separated by a human ATM intron fragment containing stop codons in all frames (RFP2GFP-ATM); successful splicing leads to an equivalent ratio of green and red fluorescence. U2OS and 293 T cells were seeded in 6 well glass bottom plates. The following day, cells were treated with 0.1, 0.5, 1 or 5 μM 8-Azaguanine. Approximately 5 hours later, cells were transfected with the RFP2GFP-ATM construct and incubated for further 48 hours. Following this, single cell RFP and GFP fluorescence intensity was acquired in 10,000 cells/well using high-content imaging and splicing was assessed as the GFP/RFP fluorescence intensity ratio relative to control/untreated cells.

### RNA-Seq

Nuclear and Cytoplasmic RNAs were separated using a hypotonic buffer. RNA-Seq libraries were prepared using the SMARTer® Stranded Total RNA Sample Prep Kit (634873 Clontech). Sequencing reads were aligned using STAR^50^, transcripts quantified using DESeq2^51^ and differential splicing analysed by dSpliceType^52^. Random subsampling analysis via RSeQC^53^ showed that the read coverage approached saturation of identified splice junctions, meaning that, statistically, all known and many of the novel junctions would have likely been identified at the coverage level achieved for all samples (Fig. S4D).

## Supplementary information

Supplemental Methods & Figures

Table S1

Table S2

Table S3

Table S4

Table S5

Table S6

Table S7

Table S8

Table S9

## Data Availability

The RNA-Seq dataset generated during the current study is available in the ArrayExpress (EMBL-EBI) repository, under accession E-MTAB-7192. The proteomics dataset generated during the current study will be made available in the ProteomeXchange repository. All other data generated and analysed during this study are included in this published article (and its Supplementary Information files).
