# Supplementary material for: Altered splicing and cytoplasmic levels of tRNA synthetases in SF3B1-mutant myelodysplastic syndromes as a therapeutic vulnerability
Source: Sci Rep. 2019 Feb 25;9:2678. doi: 10.1038/s41598-019-39591-7 (PMC6390101; doi:10.1038/s41598-019-39591-7)
Supplement: Supplementary file 1 — Supplemental Methods & Figures [file 41598_2019_39591_MOESM1_ESM.pdf]

Supplemental Methods & Figures

Altered splicing and cytoplasmic levels of tRNA synthetases in SF3B1-mutant myelodysplastic syndromes as a therapeutic vulnerability

Fabio Giuseppe Liberante[1,2*]

Katrina Lappin[1]

Eliana M Barros[1]

Jekaterina Vohhodina[1]

Florian Grebien[2,3]

Kienan I Savage[1†]

Kenneth Ian Mills[1†]

Affiliations:
1: Centre for Cancer Research and Cell Biology (CCRCB), Queen's University Belfast, Belfast, United Kingdom

2: Ludwig Boltzmann Institute for Cancer Research, Vienna, Austria

3: Institute for Medicinal Biochemistry, University of Veterinary Medicine, Vienna, Austria

* Corresponding author

† These authors contributed equally to this work

Corresponding Author:
Fabio G Liberante
Ludwig Boltzmann Institute for Cancer Research
Währinger Straße 13A
1090 Vienna
Austria
TEL: +431250774207
EMAIL: fliberante01@qub.ac.uk

Supplemental Methods

Oligos

All DNA sequences (gRNA oligos, ssODNS, primers etc.) used in the construction and screening of the cell line models are in Table S1.

CRISPR/Cas9

To generate isogenic cell line models of SF3B1 K700E mutation CRISPR/Cas9 technology was employed. A schematic at (Figure S1A) illustrates the locus that was targeted, the gRNA used and the substitutions targeted.

Guide RNAs were chosen using the online tool designed by Zhang et al (http://crispr.mit.edu/) to target within 35bp of the K700E A>G transition site in the human genome (hg38), with the lowest number of predicted off-target sites. Ultimately, the chosen gRNA (GTCCGGACCATCAGTGCTT) was only 19bp, to increase specificity[1] and to allow it to naturally start with a G residue enabling U6 driven expression.

To create a substitution at the correct nucleotide, ssODNs were designed that contained the desired substitutions together with 77bp of homology either side of the targeted substitutions. To allow RFLP screening of clones, a second synonymous substitution was targeted to the adjacent Valine codon (V701V) to create a novel MspI restriction site. This exact nucleotide position was predicted by HSF3.0[2] to have minimal impact on the nearby upstream splice junction. The ssODNS were ordered as Ultramers from IDT, resuspended in TE buffer at 100 µM and stored at -20°C.

The ssODN (100nM final concentration) together with the gRNA-expressing plasmid (1.5 µg) were introduced into the K-562 cells using the Amaxa 4D Nucleofector using the manufacturer recommended protocol. After 36h of recovery, the cells were sorted to purity for GFP-positivity using FACS (BD FACSAria II, Becton Dickson). In initial experiments, there was a high level of death in the GFP positive population (data not shown). This fits with what has been shown previously, both by our lab and others[3], in that knockdown or knockout or SF3B1 leads to cell death. Therefore, GFP^+ cells were incubated for a further 48h before dilution cloning. Surviving cells were plated at a rate of ½ a cell per well in four 384-well plates. After 2 weeks of incubation, growing clones were isolated and expanded into larger cultures.

PCR & Pyrosequencing

Clonal populations were expanded and colony PCR was used to amplify the targeted allele. Cell pellets were first heat-lysed in TE buffer at 95 °C for 3 minutes before adding the PCR master mix (1U GenScript GreenTaq in 20 uL reaction with FastDigest Green Buffer, dNTPs and primers). Cycling conditions were: 94 °C 3 min | 35 cycles [94 °C 20s | 60 °C 30s | 72 °C 45s] | 72 °C 2 min | 4 °C hold

The amplicons were subjected to restriction fragment length polymorphism (RFLP) screening to check for the creation of the MspI site by the incorporation of the synonymous V701V transition. In brief, 0.5uL Fast Digest MspI (Thermo Scientific) restriction enzyme was spiked directly into the PCR reaction, incubated for 1 hour at 37 °C and loaded directly on a 1% agarose gel. Successfully modified clones show a double band lower than the WT. In all clones screened, at least one wildtype undigested band was present (Figure S1B).

The genomic allele burden and mRNA expression level of the mutant allele was checked by Pyrosequencing. Amplicons were designed using the PyroMark Assay Design (Qiagen) software. Biotinylated primers were used to amplify genomic or complementary DNA (intron-spanning primer pairs) and amplicons purified using a PCR clean-up kit (GeneJET, Thermo Scientific). Purified amplicons were then bound, denatured and sequenced using the PyroMark q24 platform.

Primers were designed[4] to quantify the cryptic SNURF/SNRPN exon. A common forward primer was targeted to exon 2, different reverse primers were used to quantify cryptic exon 2b levels relative to exon 3. Quantitative RT-PCR was normalised using deltaCT values from expressed Alu repeat (EAR) primers, which are considered more stable than single housekeeping genes, especially in circumstances of suspected global transcriptome changes.[5]

FISH

For FISH analysis of SF3B1 copy number, growing cells were sent to Catherine Green & Daniela Moralli at the Wellcome Centre for Human Genetics, Oxford. Fosmid G248P85642F7 (WI2-1719K14) was used, which overlaps the SF3B1 region in band 2q33.1 (GRCh37/hg19 chr2:198,256,698-198,299,771). Total chromosome 2 copy number was simultaneously analysed by whole chromosome paint 2 (WCP2).

For polyA FISH, cells were cytospun on glass slides, fixed and permeabilised in 4% formaldehyde in 20% methanol. Oligo(d)T_{50} probes with 5' Cy3 fluorescent labelling were incubated with the cells and yeast tRNA used as a blocking agent, as previously described.[6] The ratio of nuclear to cytoplasmic staining was quantified using an image analysis script written in ImageJ.[6]

Immunoprecipitation, SILAC & Mass Spectrometry

Crude cellular lysates were extracted from cells using lysis with ELB (50 mM Tris pH 8.0, 50 mM NaCl, 0.5% SDS, 1 mM EDTA). M280 DynaBeads (Invitrogen) and a polyclonal SF3B1 antibody targeting the N-terminal region between amino acids 100~150 (Bethyl Labs A300-996A) were used to immunoprecipitate total SF3B1 protein. This precipitate was denatured with Visio loading buffer (Advansta) and run on an SDS-PAGE gel (4~12% Bis-Tris Invitrogen). The visualized bands were excised and frozen. The frozen gel slices were sent to FingerPrints Proteomics (Dundee) for trypsin digestion and mass spectrometry analysis.

For SILAC; mutant cells were grown in “medium” R6K4 RPMI1640 and wildtype cells in “light” RPMI1640 both supplemented with 10kDa dialyzed FBS (FingerPrints Proteomics, Dundee).

Raw Mass Spectrometry data were imported directly into MaxQuant quantitation software. Peptide spectra were mapped to the UniProt Reference Proteome (UP000005640) with the expected K700E peptide fragment prepended to the SF3B1 amino acid sequence with an XK spacer (including one extra fragment in each direction to account for missed cleavages). Raw matches from MaxQuant were filtered based on the following criteria; no reverse hits/contaminants, Q-value <0.05, unique sequence coverage ≥2%, unique peptides >1, quantifiable intensity, normalised ratios ±1.5, only first and second UniProt protein hits.

Protein synthesis assay

The EZClick™ Global Protein Synthesis Assay Kit (BioVision Inc., San Francisco, USA) was used to measure global translation rates. In brief, OP-Puromycin is added directly to cells in culture, whereby its incorporation into nascent polypeptides in a fixed time can be quantified using fluorescent click chemistry and flow cytometry. The translational inhibitor cycloheximide (CHX) was used to block protein synthesis as a negative control for specificity of staining.

Drug treatment

Cells were treated with varying doses of 8-azaguanine (sc-207194, Santa Cruz Biotechnology), dissolved in DMSO, in 96-well plates for 5 days. Final concentration of DMSO was 0.1% in all cases: untreated controls showed no detectable difference in viability. Viability was measured using CellTiterGlo (Promega) and normalized to the vehicle-treated signal as 100%.

RNA-Seq

Total/Nuclear/Cytoplasmic

Cytoplasmic RNA extraction was performed essentially as described in Gagnon et al.[7] Briefly, $1x10^7$ cells were resuspended in ice-cold hypotonic lysis buffer (HLB) supplemented with SUPERase Inhibitor (Ambion). The successful lysis of the outer cellular membrane to release cytoplasmic only RNAs was assessed visually by phase contrast microscopy, showing free, intact nuclei (Figure S2D). These free nuclei were then precipitated by centrifugation at 1000 x g for 3 minutes. The cytoplasmic supernatant was spun again at maximum speed for 2 minutes to remove any contaminating nuclei. Nuclei and cytoplasmic fractions were mixed directly with TRIzol supplemented with 1mM EDTA to inhibit ion-mediated RNA catalysis and heated at 65°C for 10 minutes. Samples were mixed vigorously with Chloroform and spun at maximum speed at 4°C for 15 minutes. The aqueous, RNA-containing, fraction was then mixed 1:1 with ethanol and applied to an RNA-binding column (DIRECTzol, Zymo Research, USA). The RNA was then washed and on-column DNAse I digested, before elution with RNase-free

water. Preparation of total cellular RNAs was performed in a similar fashion, except cells were directly lysed in TRIzol.

HLB buffer: 10 mM Tris-HCl (pH 7.5), 10 mM NaCl, 3 mM MgCl2, 0.1% (vol/vol) IGEPAL CA-630 and 10% (vol/vol) glycerol.

RNA was quantified by spectrophotometry (NanoDrop ND1000) and samples then normalised with water to 600ng/uL before further processing.

Quality control

Primers against lncRNAs NEAT1 & MALAT1 were designed (Table S1) and used to assess nuclear enrichment and cytoplasmic purity by comparison to more widely distributed mRNAs for ACTB and B2M.[8,9]

Total RNA samples were run on a Bioanalyzer chip to assess integrity was greater than RIN=8 (Agilent RNA 6000 Nano Kit).

Library preparation

Before library preparation, in order to provide controls for downstream RNA-Seq analysis, artificially synthesized non-mammalian RNA fragments (SIRVs, Lexogen, Austria) were spiked-in to purified RNA at a level of approximately 2% by mass.

Sequencing libraries were prepared from spiked total RNA using the SMARTer® Stranded Total RNA Sample Prep Kit (634873 Clontech). Briefly, RNA is ribo-depleted and reverse-transcribed with random priming; then the cDNA amplified by PCR with simultaneous addition of adapters and barcodes.

After final clean-up and removal of primer-dimers the libraries were size selected using SPRI beads (Beckman Coulter). Negative selection, with ratios of 0.7× beads & 1.2× beads, was used to remove library fragments significantly longer than ~600bp; the maximum Illumina fragment sequencing length.

Sequencing

Individual libraries were quantified using the KAPA Library Quantification Kit (KK4835, Kapa Biosystems, USA) and subsequently pooled at 4nM. The pool was then quantified again and diluted to 2nM before denaturing and spike-in with 8% phiX. The libraries were sequenced using the Illumina NextSeq platform with a 150 cycle high-output kit. The second run was set to 80-6-80 base pairs to allow for the 6bp index and maximize read length.

Analysis

Raw reads were trimmed using the Trim Galore[10] package, which makes use of the Cutadapt[11] library. Any nucleotides scoring <Q20 or containing >0 matches to the Illumina adapters were trimmed and

any trimmed reads less than 20bp were removed. Any reads remaining without mates were removed. FastQC[12] was also run on trimmed reads to check for artefacts.

Trimmed read pairs were initially aligned using STAR[13] to a human genome (hg38) index including GENCODE v23 annotations and the Lexogen SIRV spike-in sequence/annotation set. The identified splice junctions evidenced by more than 2 split reads were combined and used to build a new augmented genome index representing as many splice variants as possibly present in the K-562 cells. This augmented genome was then used as a reference for the final alignment of the RNA-Seq data.

Total read number per sample varied between 68.6~140.7 million reads, with uniquely mapped reads constituting 81~86%. Read alignment to SIRVs was between 1.6~1.7%, which corresponds closely to the 2% RNA spiked-in. The coverage of each artificial transcript was consistent with the defined ratios. Furthermore, as they contain different "splice variants", using an intentionally incomplete exon annotation file allows testing of whether novel splice junctions are successfully identified by the analysis pipeline. In all samples all SIRV splice junctions were successfully identified.

Read counts per gene were generated using the <ReadsPerGene> function of STAR. Read count-based differential gene expression was carried out using the DESeq2[14] algorithm with VST normalization via RNA-Seq2G.[15] Differential-splicing and -expression analysis was executed using the dSpliceType algorithm.[16] This required generation of junction bed files from the bam files using regtools (https://github.com/griffithlab/regtools). Splicing and read coverage was analysed against the ENSEMBL hg38 v84 annotation.

In order investigate gene fusions or trans-splicing, the chimeric alignment reads identified by STAR-Fusion were converted back to FASTQ format and re-mapped using the JAFFA fusion-finding pipeline[17] against the hg38 GENCODE v20 transcript database. This tool identifies reads or read pairs that span more than one gene either within or between chromosomes. It only identified one expressed fusion transcript in any of the samples, with both high confidence and greater than 5 spanning reads. Both SF3B1 wildtype and mutant cells harboured the t(9;22)BCR:ABL translocation and reciprocal NUP214:XKR3, both known translocations in the K-562 cell line.

Gene Set Enrichment Analysis

Identified gene lists were assessed for enrichment using the online Enrichr[18] platform. The gene interaction networks were built and annotated through Cytoscape[19] using the STRING-DB[20] interaction database. Proportional overlap Venn diagrams were generated using BioVenn.[21]

References

1. Fu, Y., Sander, J. D., Reyon, D., Cascio, V. M. & Joung, J. K. Improving CRISPR-Cas nuclease specificity using truncated guide RNAs. *Nat. Biotechnol.* **32,** 279–84 (2014).
2. Desmet, F.-O. *et al.* Human Splicing Finder: an online bioinformatics tool to predict splicing signals. *Nucleic Acids Res.* **37,** e67 (2009).
3. Zhou, Q. *et al.* A Chemical Genetics Approach for the Functional Assessment of Novel Cancer Genes. *Cancer Res.* **75,** 1949–1958 (2015).
4. Ye, J. *et al.* Primer-BLAST: A tool to design target-specific primers for polymerase chain reaction. *BMC Bioinformatics* **13,** 134 (2012).
5. Marullo, M. *et al.* Expressed Alu repeats as a novel, reliable tool for normalization of real-time quantitative RT-PCR data. *Genome Biol.* **11,** R9 (2010).
6. Vohhodina, J. *et al.* The RNA processing factors THRAP3 and BCLAF1 promote the DNA damage response through selective mRNA splicing and nuclear export. *Nucleic Acids Res.* **45,** 12816–12833 (2017).
7. Gagnon, K. T., Li, L., Janowski, B. a & Corey, D. R. Analysis of nuclear RNA interference in human cells by subcellular fractionation and Argonaute loading. *Nat. Protoc.* **9,** 2045–60 (2014).
8. Cabili, M. N. *et al.* Localization and abundance analysis of human lncRNAs at single-cell and single-molecule resolution. *Genome Biol.* **16,** 20 (2015).
9. van Heesch, S. *et al.* Extensive localization of long noncoding RNAs to the cytosol and mono- and polyribosomal complexes. *Genome Biol.* **15,** R6 (2014).
10. Krueger, F. Trim Galore. *A wrapper tool around Cutadapt and FastQC to consistently apply quality and adapter trimming to FastQ files* (2012). Available at: http://www.bioinformatics.babraham.ac.uk/projects/trim_galore/.
11. Martin, M. Cutadapt removes adapter sequences from high-throughput sequencing reads. *EMBnet.journal* **17,** 10 (2011).
12. Andrews, S. FastQC: A quality control tool for high throughput sequence data. (2010). Available at: http://www.bioinformatics.babraham.ac.uk/projects/fastqc/.
13. Dobin, A. *et al.* STAR: ultrafast universal RNA-seq aligner. *Bioinformatics* **29,** 15–21 (2013).
14. Love, M. I., Huber, W. & Anders, S. Moderated estimation of fold change and dispersion for RNA-seq data with DESeq2. *Genome Biol.* **15,** 550 (2014).
15. Zhang, Z., Zhang, Y., Evans, P., Chinwalla, A. & Taylor, D. RNA-Seq 2G: Online Analysis Of Differential Gene Expression With Comprehensive Options Of Statistical Methods. *bioRxiv* (2017).
16. Zhu, D., Deng, N. & Bai, C. A generalized dSpliceType framework to detect differential splicing and differential expression events using RNA-Seq. *IEEE Trans. Nanobioscience* **14,** 192–202 (2015).
17. Davidson, N. M., Majewski, I. J. & Oshlack, A. JAFFA: High sensitivity transcriptome-focused fusion gene detection. *Genome Med.* **7,** 43 (2015).
18. Kuleshov, M. V *et al.* Enrichr: a comprehensive gene set enrichment analysis web server 2016 update. *Nucleic Acids Res.* **44,** W90-7 (2016).
19. Shannon, P. *et al.* Cytoscape: a software environment for integrated models of biomolecular interaction networks. *Genome Res.* **13,** 2498–504 (2003).
20. Jensen, L. J. *et al.* STRING 8--a global view on proteins and their functional interactions in 630 organisms. *Nucleic Acids Res.* **37,** D412–D416 (2009).
21. Hulsen, T., de Vlieg, J. & Alkema, W. BioVenn - a web application for the comparison and visualization of biological lists using area-proportional Venn diagrams. *BMC Genomics* **9,** 488 (2008).

Supplemental Figure Legends

Figure S1A
A schematic illustrating the genomic locus at the intron 15 - exon 16 boundaries (ENST00000335508.10|NM_012433.3) that was targeted with CRISPR/Cas9. The splice junction, K700E substitution, V701V synonymous restriction site creation and gRNA target are all marked.

Figure S1B
Restriction fragment length polymorphism (RFLP) screening (MspI) of PCR-amplified genomic DNA from a representative set of isolated clones. Ladder is 100bp (NEB, Germany).

Figure S1C
Sanger chromatogram from three different bacterial clones of DNA cloned from the targeted genomic region of the SF3B1$^{+/\Delta/\Delta}$ cell clone. One allele shows wildtype sequence, the other two show 14bp and 28bp deletions respectively.

Figure S1D
Photographic image of SDS-PAGE of immunoprecipitated SF3B1 protein (Bethyl Labs A300-996A), stained with Coomassie blue.

Figure S2A
Plot of fold enrichment of nuclear extract MALAT1/NEAT1 lncRNA levels over cytoplasmic extracts normalized to the geometric mean of ACTB and B2M levels by qPCR (Error bars represent SEM, n=2, technical triplicate).

Figure S2B
RNA-Seq, total read count-normalized, coverage plots at the SNORD47, MALAT1 and NEAT1 genomic loci for the nuclear and cytoplasmic RNA preps. All within group scales are set equal to allow comparison.

Figure S2C
WebLogo showing base composition of nucleotide sequence at positions -29 – +3 of splice junctions that were alternatively-spliced according to RNA-seq analysis of tRNA synthetase mRNAs.

Figure S2D
KEGG Aminoacyl-tRNA biosynthesis pathway (hsa00970). Cytoplasmic depleted nodes are blue and increased red.

Figure S2E
Relative quantity real-time PCR for WARS in nuclear or cytoplasmic RNA fractions. *p-value <0.05 ***p-value <0.0001

Figure S3A
Scatter plot of log2-transformed protein fold change (SILAC n=1) vs whole cell transcript fold change (RNA-Seq - DESeq2, n=2) Pearson correlation = -0.008

Figure S3B
Scatter plot of log2-transformed fold changes for all 3 pairwise comparisons of total, nuclear and cytoplasmic RNA-Seq data (DESeq2, n=2). tRNA synthetase mRNAs highlighted in blue.

Figure S4A
Cell pellets - WT vs Mutant (K700E) showing visible difference in red colour.

Figure S4B
Unlabelled and cycloheximide treated cells represent negative controls for OP-Puro staining. Cycloheximide blocks ribosome function and incorporation of OP-Puro. Table represents mean fluorescence intensity (MFI).

Figure S4C
Fluorescence microscopy of 293T cells transfected with the homologous recombination reporter plasmid pmKaxxte harbouring a ~1.5kb fragment of genomic DNA centred around the gRNA target site. Successful targeting of the plasmid by the SF3B1 gRNA will result in recombination and restoration of the mKate2.5 ORF and red fluorescence.

Figure S4D

RSeQC Plot of random read sub-sampling plotted against number of junctions identified in sampled reads. Plotted for both known junctions and novel junctions. Plateau implies saturation of junction identification.

Figure S4E

Phase microscopy image of cells before and after membrane lysis with HLB buffer.

Figure S4F

Full gel image of Figure 2C - RT-PCR of cDNA from wildtype, SF3B1^{K700E} & SF3B1$^{+/\Delta/\Delta}$ whole cell (T), nuclear (N) and cytoplasmic fractions (C). Amplicon spans ABCB7 exons 8-9 (RefSeq NM_004299).

Table S1

List of DNA sequences used in study; including ssODN, cloning oligos, RFLP-PCR and qPCR primers

Table S2

Differentially-spliced genes comparing SF3B1^{K700E} mutant to SF3B1 wildtype MDS patient CD34+ bone marrow cells from Dolatshad et al. using dSpliceType (4 mutant & 4 wildtype patients).

Table S3

Differentially-spliced genes comparing SF3B1^{K700E} mutant to SF3B1 wildtype NALM-6 cells from Darman et al. using dSpliceType (n=3).

Table S4

Differentially-spliced genes comparing SF3B1^{K700E} mutant to SF3B1 wildtype K-562 cells from this study using dSpliceType (n=2).

Table S5

Results of MaxQuant analysis of raw Mass Spectrometry SILAC data, using standard thresholds and mapped to a modified UniProt Human Reference Proteome (UP000005640) (n=1).

Table S6

Differentially-expressed genes comparing SF3B1^{K700E} mutant to SF3B1 wildtype MDS patient CD34+ bone marrow cells from Dolatshad et al. using DESeq2 with VST normalization (4 mutant & 4 wildtype patients).

Table S7

Differentially-expressed genes comparing SF3B1^{K700E} mutant to SF3B1 wildtype NALM-6 cells from Darman et al. using DESeq2 with VST normalization (n=3).

Table S8

Differentially-expressed genes comparing SF3B1^{K700E} mutant to SF3B1 wildtype K-562 cells from this study using using DESeq2 with VST normalization, both total and cytoplasmic RNAs (n=2).

Table S9

Quantity of tRNA synthetases (KEGG hsa00790) at whole-cell, nuclear, cytoplasmic RNA and protein levels.

Figure S1

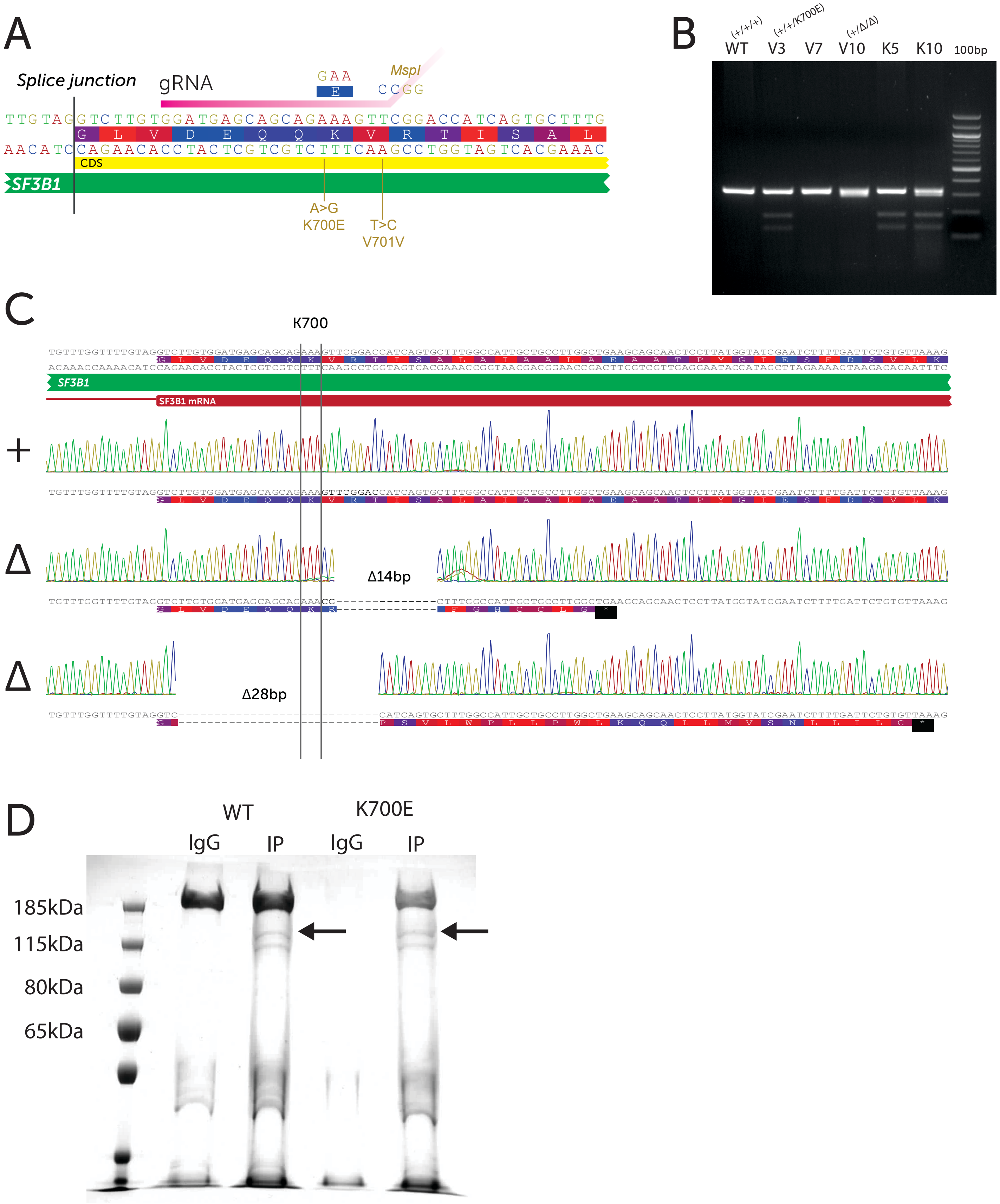

IP -> LFQ-Mass Spec
Peptide ratio in mutant cells
Lysine (K) (53%)
Glutamic Acid (E) (46%)

Figure S2

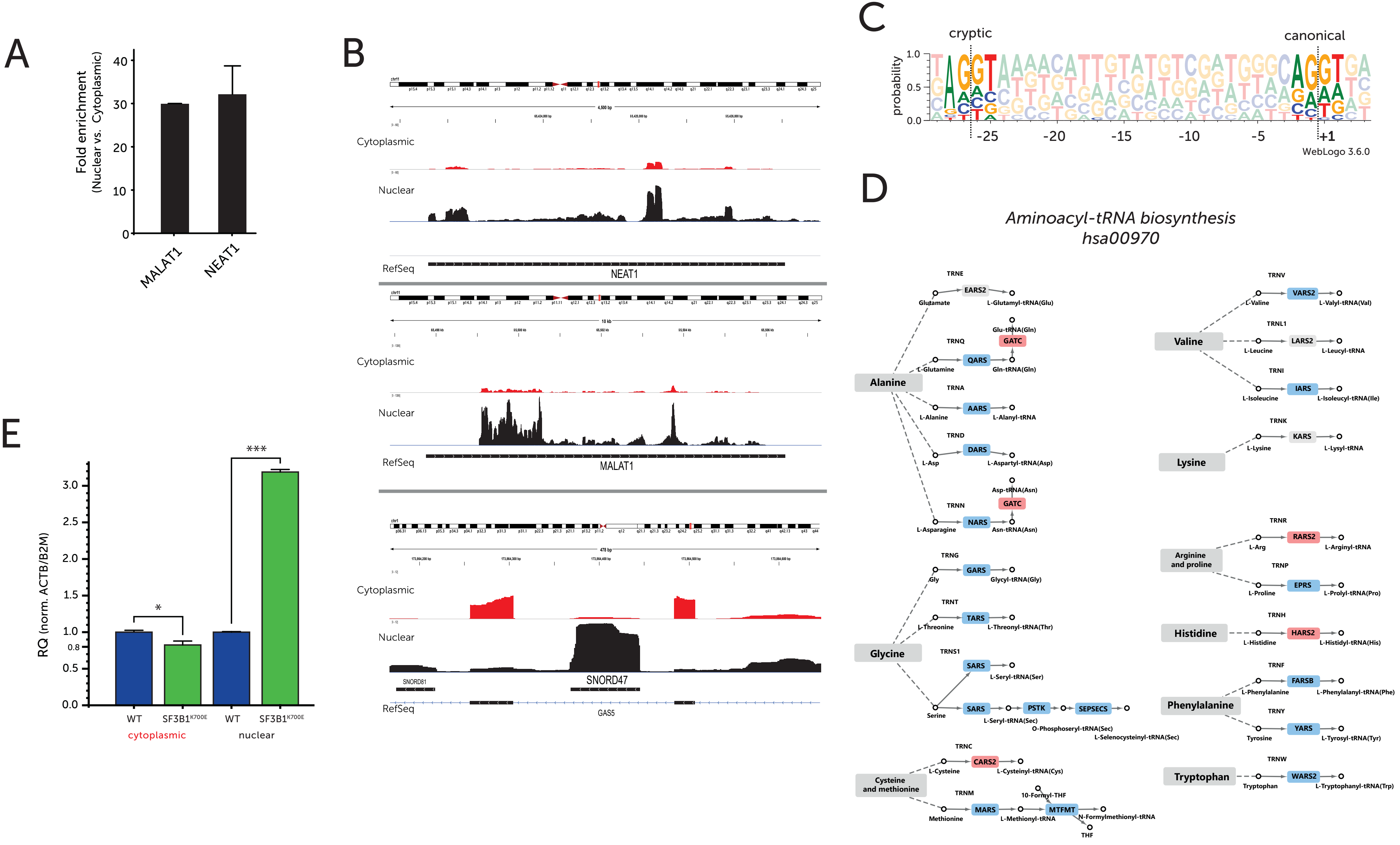
A
Fold enrichment (Nuclear vs. Cytoplasmic)
MALAT1
NEAT1
B
Cytoplasmic
Nuclear
RefSeq
NEAT1
MALAT1
SNORD81
SNORD47
GAS5
C
cryptic
canonical
probability
WebLogo 3.6.0
D
Aminoacyl-tRNA biosynthesis
hsa00970
Alanine
Glycine
Cysteine and methionine
Valine
Lysine
Arginine and proline
Histidine
Phenylalanine
Tryptophan
E
RQ (norm. ACTB/B2M)
WT
SF3B1K700E
cytoplasmic
nuclear

Figure S3

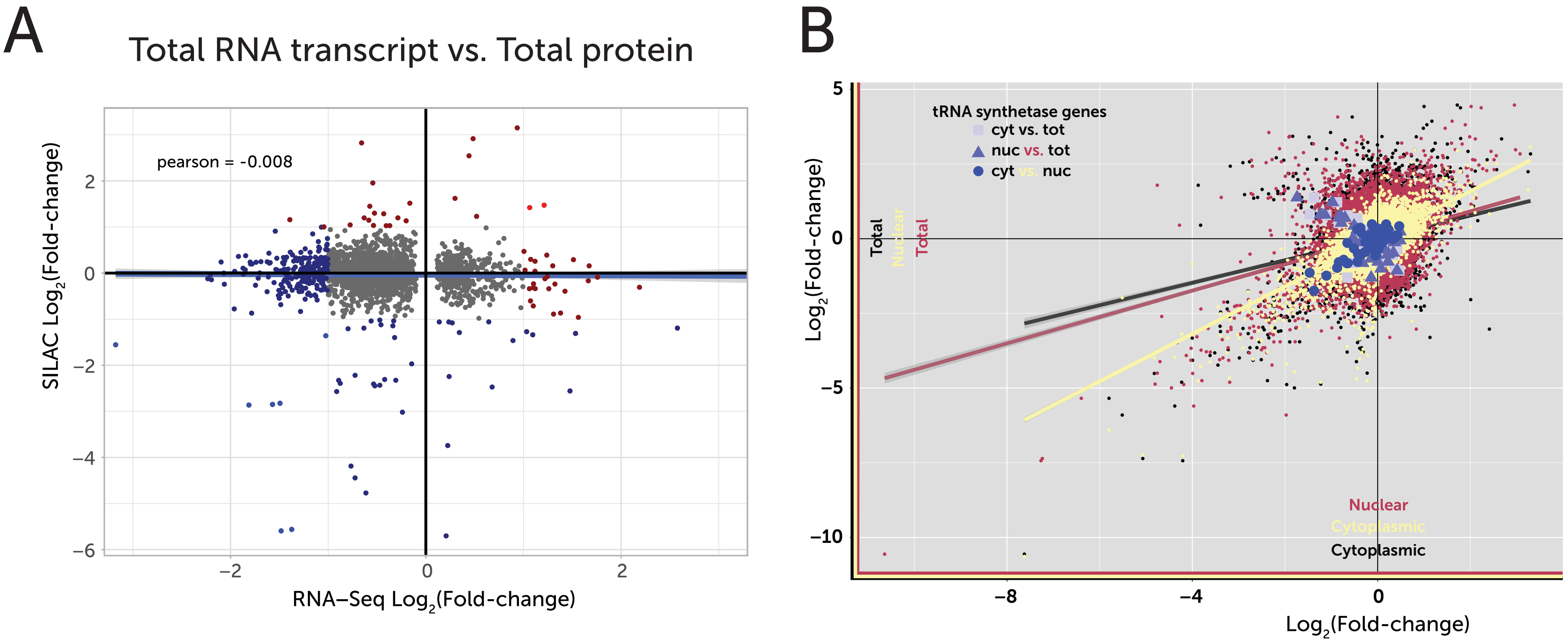
A
Total RNA transcript vs. Total protein
pearson = -0.008
SILAC Log2(Fold-change)
RNA–Seq Log2(Fold-change)
B
tRNA synthetase genes
cyt vs. tot
nuc vs. tot
cyt vs. nuc
Total
Nuclear
Total
Log2(Fold-change)
Nuclear
Cytoplasmic
Cytoplasmic
Log2(Fold-change)

Figure S4

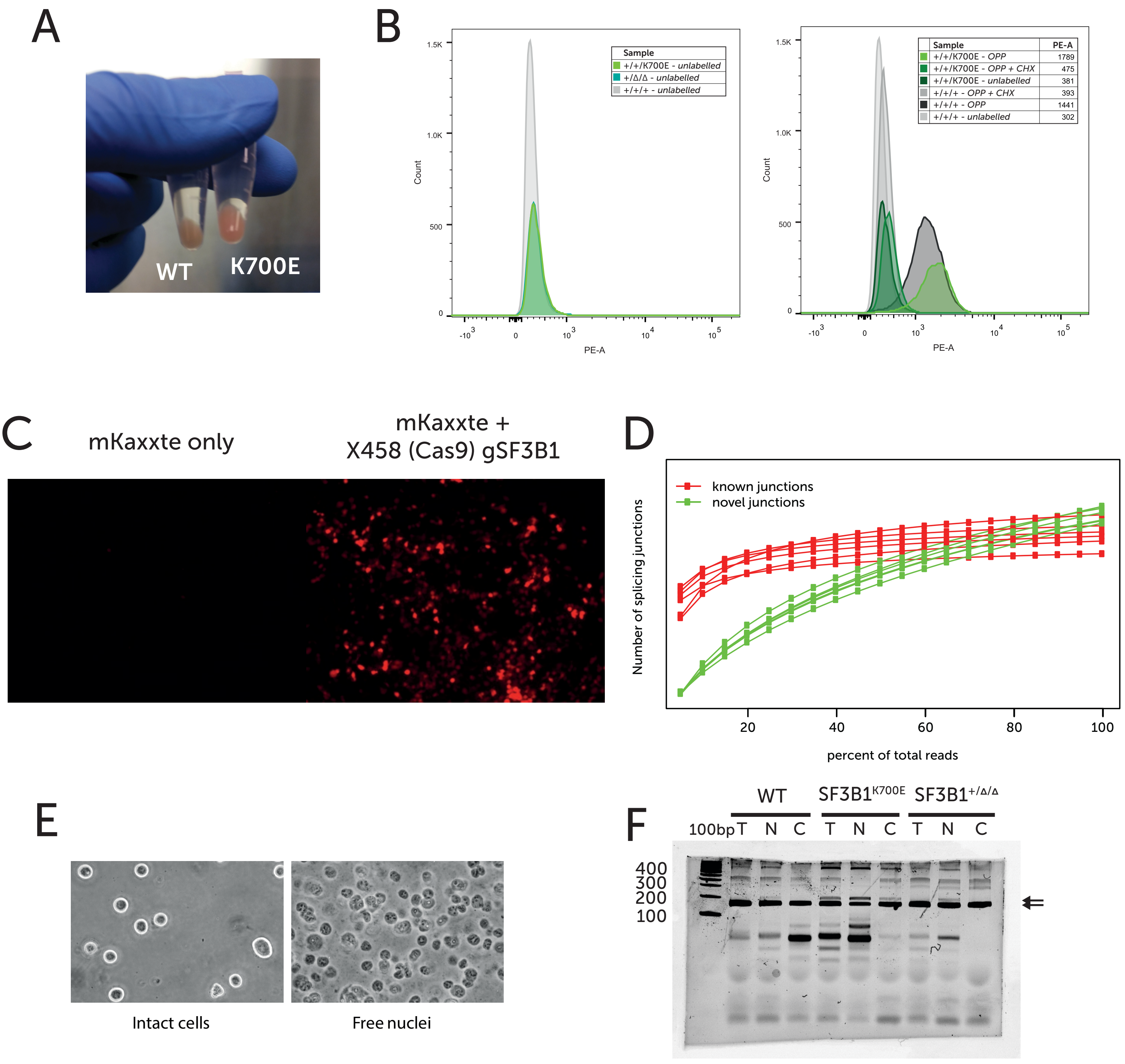
A
WT
K700E
B
Sample
+/+/K700E - unlabelled
+/Δ/Δ - unlabelled
+/+/+ - unlabelled
Count
PE-A
Sample
PE-A
+/+/K700E - OPP
1789
+/+/K700E - OPP + CHX
475
+/+/K700E - unlabelled
381
+/+/+ - OPP + CHX
393
+/+/+ - OPP
1441
+/+/+ - unlabelled
302
C
mKaxxte only
mKaxxte +
X458 (Cas9) gSF3B1
D
known junctions
novel junctions
Number of splicing junctions
percent of total reads
E
Intact cells
Free nuclei
F
WT
SF3B1K700E
SF3B1+/Δ/Δ
100bp
T N C T N C T N C
400
300
200
100
